# Characteristics and clinical outcomes of COVID‐19 patients with pulmonary disorders: A single‐center, retrospective observational study

**DOI:** 10.1002/hsr2.525

**Published:** 2022-02-22

**Authors:** Wasim Jamal, Mohamad Y. Khatib, Mohammad Al Wraidat, Amna Ahmed, Dore C. Ananthegowda, Ahmed S. Mohamed, Asra Aroos, Prem Chandra, Mansoor Hameed, Muhammad Yousaf, Ahmed Al‐Mohammed, Abdulqadir J. Nashwan

**Affiliations:** ^1^ Department of Critical Care Hazm Mebaireek General Hospital (HMGH), Hamad Medical Corporation (HMC) Doha Qatar; ^2^ College of Medicine Weill Cornell Medicine‐Qatar Ar‐Rayyan Qatar; ^3^ Academic Health System, Hamad Medical Corporation (HMC) Doha Qatar; ^4^ Department of Medicine Hamad General Hospital (HGH), Hamad Medical Corporation (HMC) Doha Qatar

**Keywords:** asthma, coinfection, COVID‐19, lung disease

## Abstract

**Introduction:**

Mortality rates and clinical characteristics of patients with coronavirus disease 2019 (COVID‐19) admitted to the intensive care unit (ICU) vary significantly.

**Objectives:**

To describe the data of patients with pulmonary comorbidities who were admitted to the ICU with COVID‐19 in Qatar in terms of demographic characteristics, coexisting conditions, imaging findings, and outcomes.

**Methods:**

We conducted a retrospective study of the outcomes with regard to mortality and requirement of invasive ventilation, demographic characteristics, coexisting conditions, secondary infections, and imaging findings for critical care patients with COVID‐19 in Qatar who had pulmonary comorbidities between March and June 2020.

**Results:**

A total of 923 patients were included, 29 (3.14%) were found to have pulmonary disease. All these 29 patients' respiratory disease was noted to be asthma. Among these, three patients (10.3%) died in the ICU within 28 days of ICU admission. They were all above 50 years old. Nineteen (66%) patients required intubation and mechanical ventilation. Twenty‐one (72.4%) patients were males. The most common comorbidities included diabetes mellitus (55.1%) and hypertension (62%). Eighteen (62%) patients developed secondary infections in the ICU. Five (17.24%) patients developed renal impairment. Twenty (69%) patients received tocilizumab as part of their COVID‐19 management, and out of these 16 (80%) patients developed a coinfection.

**Conclusion:**

Patients with pulmonary disorders had higher mortality rates than other patients admitted to ICU during the same time frame with similar comorbidities; these patients require extra consideration and care to avoid disease progression and death.

## 1 INTRODUCTION

Since coronavirus disease 2019 (COVID‐19) was discovered, which is caused by severe acute respiratory syndrome coronavirus‐2 (SARS‐CoV‐2). It swiftly spread, resulting in an epidemic throughout the globe.^1^, ^2^ While most patients have mild symptoms, some people experience acute respiratory distress syndrome (ARDS), possibly triggered by septic shock, multiorgan failure, cytokine storm, and blood vessels thrombosis.^3^, ^4^ Disease outcomes varied across various regions and countries. While increasing age was considered a significant risk factor,^3^ comorbidities and coexisting medical conditions proved critical other factors in determining the severity of the disease and mortality outcomes.^5^, ^6^, ^7^ It subsequently and steadily became more relevant and essential to determine the effect of various diseases on COVID‐19 outcomes. Over the course of the pandemic, evidence emerged that certain medical conditions could influence the outcomes more than the other disease. In relation to this, we focused on outcomes in COVID‐19 patients who have known respiratory conditions—we conducted a retrospective study of the outcomes with regard to mortality and requirement of invasive ventilation, demographic characteristics, coexisting conditions, secondary infections, and imaging findings for critical care patients with COVID‐19 in Qatar who had pulmonary comorbidities between March 21, 2020, and June 14, 2020.

## 2 MATERIALS AND METHODS

A retrospective observational study was conducted to describe the characteristics of COVID‐19 patients who were admitted to the intensive care unit (ICU) in Hazm Mebaireek General Hospital (HMGH), a member of Hamad Medical Corporation (the primary healthcare provider in Qatar). The data were collected from the patients' electronic medical records (Cerner). All consecutive 18 years or older patients with confirmed SARS‐CoV‐2 infection (laboratory) were admitted to the ICU in HMGH between March 21, 2020, and June 14, 2020, and had a confirmed diagnosis of a pulmonary disease documented in the patient medical record were included.

### 2.1 Statistical analysis

Data were analyzed using Microsoft Excel 2010 (Microsoft, Redmond, Washington) for Windows. All appropriate descriptive statistics were utilized to summarize and describe the study variables, such as frequencies and percentages.

## 3 RESULTS

**FIGURE 1:**
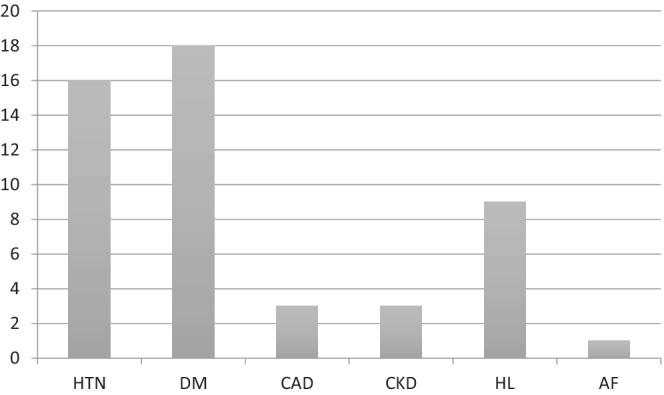
Other comorbidities of patients admitted to ICU with COVID‐19 and known pulmonary disease

**FIGURE 2:**
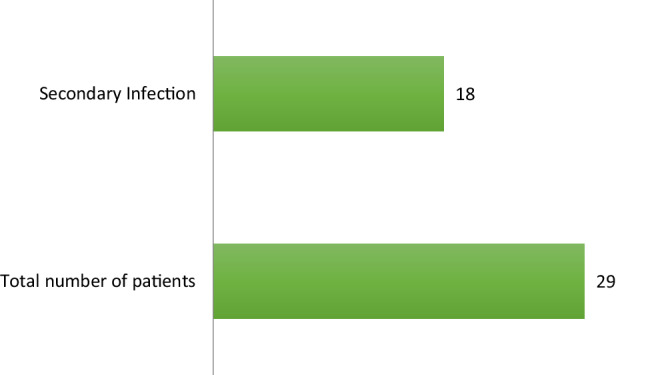
Secondary infection in confirmed COVID‐19 patients with a known pulmonary disease requiring ICU admission

From March 21 to June 14, 2020, a total of 923 patients were admitted with COVID‐19 infection to the ICU in HMGH. Out of these patients, 29 (3.14%) were found to have a pulmonary disease. All of their electronic records revealed that they had a previous diagnosis of asthma. Out of these, three (10.34%) had a smoking history. Twenty‐one (72.4%) patients were males, and 8 (27.6%) were females. The youngest patient was 33 years old, while the eldest one was 72 years old. The median age was 58 years. Twenty (69%) patients required intubation and mechanical ventilation. The most common comorbidities included diabetes mellitus type 2 (55.1%) and hypertension (62%) (Table 1). Eighteen (62%) patients developed a secondary infection in the ICU. Five patients (17.24%) developed renal impairment. Nineteen (66%) patients received tocilizumab for their COVID‐19 management; 16 of them had developed an infection following the tocilizumab injection. Three patients (10.3%) died within 28 days of ICU admission; they were all above 50 years old (Age 51, 57, and 69 Years). Out of three patients who died, two of them had hypertension (Table 2), and all three have developed secondary infections during their stay in the ICU (Figures 1 and 2).

**TABLE 1:** 28‐day mortality and invasive ventilation requirement of laboratory‐confirmed COVID‐19 patients with known pulmonary disease, by age, and gender

	All COVID‐19 patients with known respiratory disease	Age (years) Below 50	Age (years) 50 and above	Male	Female
N	29	7	22	21	8
Mortality N (%)	3 (10.34%)	0 (0%)	3 (13.6%)	3 (14.3%)	0 (0%)
Need of ventilation N (%)	19 (65.5%)	2 (28.6%)	19 (86.3%)	13 (62%)	6 (75%)

**TABLE 2:** 28‐day mortality and invasive ventilation requirement of laboratory‐confirmed COVID‐19 patients with known pulmonary disease, by comorbidities, and smoking history

	DM (n = 18)	HTN (n = 16)	CAD (n = 3)	CKD	Smoking
Mortality n (%)	0 (0%)	2 (12.5%)	0 (0%)	1 (33.3%)	0 (0%)
Requirement for ventilation n (%)	12 (66.7%)	12 (75%)	2 (66.7%)	3 (100%)	2 (66.7%)

## 4 DISCUSSION

The mortality rates of patients with COVID‐19 admitted to the ICU vary significantly in the published literature. Multiple factors explain the wide variability, such as the country's age structure, different thresholds for hospitalization, the robustness of a regional healthcare system, and availability of ICU beds, are various important factors.^8^, ^9^ Comorbidities such as diabetes mellitus,^6^ hypertension,^10^ obesity, pulmonary (such as chronic obstructive pulmonary disease (COPD)), and cardio‐cerebrovascular disease have been observed to be the more significant risk factors in patients with COVID‐19.^11^ In our single‐center, retrospective, observational study, we have presented various outcomes, including mortality and need for ventilation for COVID‐19 patients with confirmed respiratory disease diagnoses who were admitted to HMGH (a designated COVID‐19 facility in Qatar) from March to June 2020. Among the Gulf Cooperation Council (GCC) states, the infection rate is the second‐highest (December 19, 2020) in Qatar (>10 000 cases per million population).^12^ However, the overall mortality rate remained low. Qatar initiated stringent control measures to limit the spread of infection earlier during the course of the pandemic. In the wake of the COVID‐19 pandemic; Hamad Medical Corporation; the largest governmental healthcare provider in Qatar, ramped up a proactive plan to significantly expand hospital capacity, upskilling healthcare providers and involved reallocating existing resources and facilities and redeploying workforce as part of a strategy to expand critical care beds' capacity rapidly.

Pulmonary disease has been identified to affect the outcomes of patients with COVID‐19. In particular, COPD has been identified to be associated with poor outcomes.^13^, ^14^, ^15^ Smoking is also noted to be related to poor outcomes.^14^ In our cohort of patients with known respiratory disease, 28‐day mortality was 10.34%. In comparison, the total number of patients admitted to ICU during the same time frame was 923, out of which 43 patients died, giving overall 28‐day mortality of 5.6%. This data are not matched and cannot conclude an association; however, we have observed higher mortality in our cohort of patients compared to all other patients admitted to ICU during the same time course and had similar comorbidities.

Interestingly, we note that in all of our 29 patients, the respiratory disease was asthma, and there were no patients who had COPD. Asthma, being the diagnosis in all our patients, is likely multifactorial. In the Gulf States, asthma has a higher prevalence. In addition, as the diagnosis was mostly clinical and lacked adequate objective assessment, our patients were likely overdiagnosed and overpresented. Furthermore, anecdotal data suggest that overdiagnosis of asthma universally is approximately 30%.^16^, ^17^ Asthma has also been noted as common comorbidity among patients admitted to the hospital with COVID‐19. Garg et al reported that asthma was present in approximately 17% of the COVID‐19 patients admitted to the hospital, making it the fourth most prevalent comorbidity (behind hypertension, obesity, and diabetes).^18^ Not a single patient had a previous diagnosis of COPD, which is surprising; however, a few factors that may explain it at least partially is the age structure of our cohort of patients. COPD is primarily diagnosed in over 45 years old patients. Most of our patients were younger, and only three had a previous smoking history. In addition, as the diagnosis requires an objective assessment by spirometry, it is likely an underdiagnosed condition in our cohort of patients.

Although a multicenter retrospective study from the United States pertaining to hospitalized patients reported comparable mortality of 13.5% in asthmatic patients with COVID‐19, it did not find any significant mortality difference between their asthmatics and nonasthmatics patients.^19^ However, our study only included ICU patients whose mortality is expected to be high. The lower mortality in our cohort of patients is likely multifactorial, including our patients' younger age.

As observed in other studies,^8^, ^18^ 44 diabetes mellitus (n = 18, 62%) and hypertension (n = 16, 55%) were also noted to be the commonest comorbidities among our patients (Table 3). Among the three patients in our study who died, in addition to asthma, two also had hypertension, and one had chronic kidney disease.

**TABLE 3:** 28‐day mortality and invasive ventilation requirement of laboratory‐confirmed COVID‐19 patients with known pulmonary disease, by comorbidities, and smoking history

	DM (n = 18)	HTN (n = 16)	CAD (n = 3)	CKD	Smoking
Mortality n (%)	0 (0%)	2 (12.5%)	0 (0%)	1 (33.3%)	0 (0%)
Requirement for vent n (%	12 (66.7%)	12 (75%)	2 (66.7%)	3 (100%)	2 (66.7%)

The need for invasive ventilation remained common throughout the pandemic. Numerous factors, including the healthcare system's robustness and variation in the threshold for ICU admission and intubation, can cause wide variation in the number of patients subjected to intubation and mechanical ventilation across various countries. In our cohort of patients, 66% (n = 19) required invasive ventilation to manage their COVID‐19 disease. The average duration of mechanical ventilation was 10 days. A study published in the Journal of the American Medical Association (JAMA) found a 47% intubation rate for patients who were admitted to the ICU.^20^ While presenting its outcomes related to COVID‐19 in the New York City area, a case series published in JAMA found that 12.2% of hospitalized patients required intubation and ventilation.

In comparison, 14.2% of hospitalized patients required intubation and ventilation ICU care.^21^ However, in our study, the 66% intubation requirement is for ICU admissions. In another study published in JAMA, out of 21 patients admitted to ICU, 71% required mechanical ventilation.^22^

A systematic review and data analysis regarding hospital and ICU stay comprising of 52 studies, 46 studies from China published in September 2020 reported median ICU stay of COVID‐19 patients for 8 days (IQR 5‐13 days) in China and 7 days (IQR 4‐11 days) outside of China.^23^

In our cohort of patients with respiratory comorbidities, the median ICU stay was 12 days, while the median duration of ventilation was 6 days. The median duration of ventilation for the patient who died was 9 days. A study published lately^19^ reported that although in COVID‐19 patients with asthma as a comorbidity was significantly associated with a higher rate of endotracheal intubation, mechanical ventilation, and longer hospital length of stay, it was not associated with a higher rate of ICU admission, ARDS, or death among COVID‐19 patients.

Severe acute kidney injury (AKI) is frequent in patients with COVID‐19 and ARDS and is associated with high short‐term mortality.^24^ In our cohort of patients with a known respiratory disease, 5/29 (17.24%) patients developed AKI. In the ICU settings, the development of AKI is multifactorial contributed by pathological processes, including hypotension, shock, ischemia, and the contribution from drugs' adverse effects. In addition, the cytopathic effect of the virus is likely to have a role in contributing to worsening renal functions. A European multicenter retrospective observational study reported that among 211 patients, 55 (26%) developed Kidney Disease Improving Global Outcomes (KDIGO) stage 3 AKI within 7 days after ICU admission.^24^ A large multicenter prospective cohort study on the epidemiology of AKI in ICU patients in Beijing, China, showed that KDIGO stage 3 AKI accounted for 16% of critically ill patients.^25^ Findings in these studies were comparable to our observations, albeit our cohort of patients were selected based on known respiratory disease.

**FIGURE 3:**
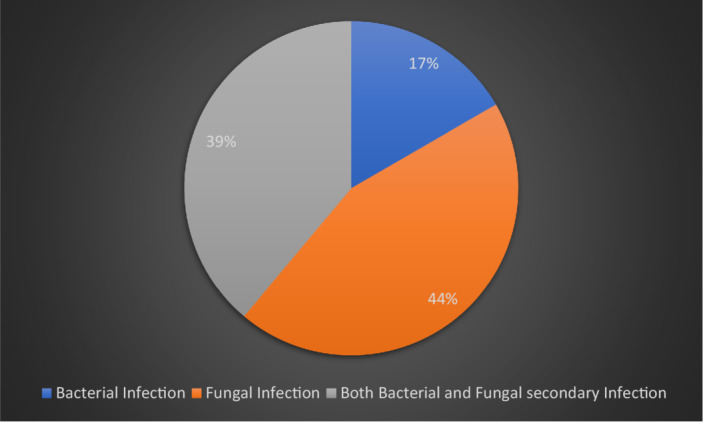
Microorganisms‐causing secondary infections

Eighteen (62%) of our patients developed a secondary infection (Table 3, Figure 3). A total of 10/18 of these patients had an infection with resistant organisms. All three patients who died developed a secondary infection during their ICU stay. Studies have so far shown an increased rate of secondary infection in patients admitted to hospital and ICU with COVID‐19 disease. Zhang et al reported 22/38 (57.89%) patients developing secondary infection with COVID‐19 in their multicenter cohort study in China 18, while Bogossian et al reported 33% multidrug‐resistant bacteria (MDRB) acquisition during ICU stay for 72 patients.^26^ Viral sepsis contributing to immunosuppression and dysregulated immune response in the face of COVID‐19 infection are among the reasons making patients more susceptible to infections. Using invasive ventilation devices was also a risk factor for developing secondary infections.^27^ Drug‐induced immunosuppression also plays a role in making patients susceptible to secondary infections. Pettit et al reported significantly increased late‐onset infections among those receiving tocilizumab,^28^ such as cytomegalovirus colitis.^29^ Kimmig et al^30^ also reported a significantly higher rate of secondary infections and mortality in their cohort of patients who received tocilizumab.

Although our data cannot conclude a causal relation between tocilizumab and the development of secondary infections, also the development of secondary infection is multifactorial; in our cohort of patients, 16 (80%) patients who had tocilizumab developed a secondary infection, and 87% had it within 10 days of tocilizumab administration.

In our cohort of 29 patients, 28 were found to have an abnormal chest X‐ray (CXR) while in the ICU. The most common pattern was bilateral infiltrates and patchy consolidation. One patient's CXR was clear, and the reason for admission to the ICU was hypotension secondary to diarrhea. In addition to patchy consolidation and bilateral infiltrates, one patient also had bilateral pleural effusion, which was likely secondary to coexistent heart failure. CXR findings in our study were consistent with other studies. Wong et al^31^ reported that the common radiological findings in his retrospective study in Hong Kong include consolidation and ground glass opacities, with bilateral, peripheral, and lower zone distributions.

## 5 CONCLUSIONS

Respiratory disease has been identified to affect COVID‐19 outcomes. In our single‐center, retrospective, observational study, we observed higher mortality in our cohort of patients with respiratory disease in comparison to all other patients admitted to ICU during the same time course and had similar comorbidities. All our patients' respiratory disease was asthma. The data were not matched and cannot conclude an association between asthma and higher mortality; however, these patients require extra consideration and care to avoid disease progression and death. Further studies are encouraged to study this association. We also observed that more than half of our patients developed secondary infections, especially those who received tocilizumab. A significant number of patients developed secondary infections with resistant organisms. A high rate of secondary infections has been noted in other studies, and we reiterate the absolute importance of measures to prevent them, strict adherence to antimicrobial stewardship, and the use of tocilizumab in carefully selected patients.

## FUNDING

This study was funded by the MRC IRB in Hamad Medical Corporation (MRC‐01‐20‐532).

## CONFLICT OF INTEREST

The authors declare that they have no competing interests.

## AUTHORS' CONTRIBUTIONS

Conceptualization: Abdulqadir Nashwan, Amna Ahmed, Wasim Jamal.

Data Curation: Abdulqadir Nashwan, Amna Ahmed, Prem Chandra, Ahmed Soliman Mohamed, Mohammad Al Wraidat, Asra Aroos, Mansoor Hameed, Muhammad Yousaf.

Methodology: Abdulqadir Nashwan, Wasim Jamal, Prem Chandra, Ahmed Soliman Mohamed.

Formal Analysis: Prem Chandra.

Supervision: Mohamad Khatib.

Validation: Mohamad Khatib.

Writing – Original Draft Preparation: Abdulqadir Nashwan, Amna Ahmed, Wasim Jamal, Prem Chandra, Ahmed Soliman Mohamed, Mohammad Al Wraidat, Asra Aroos, Mansoor Hameed, Muhammad Yousaf, Mohamad Khatib, Ahmed Al‐Mohammed, Dore Ananthegowda.

Writing – Review & Editing: Abdulqadir Nashwan, Amna Ahmed, Wasim Jamal, Prem Chandra, Ahmed Soliman Mohamed, Mohammad Al Wraidat, Asra Aroos, Mansoor Hameed, Muhammad Yousaf, Mohamad Khatib, Ahmed Al‐Mohammed, Dore Ananthegowda.

All authors read and approved the final manuscript. The first author confirm that he had full access to all of the data in the study and takes complete responsibility for the integrity of the data and the accuracy of the data analysis.

## ETHICS APPROVAL AND CONSENT TO PARTICIPATE

The project has been approved (exempted) by the Medical Research Center (MRC) IRB in Hamad Medical Corporation (MRC‐01‐20‐532). The study has been conducted in accordance with the ethical standards noted in the 1964 Declaration of Helsinki and its later amendments or comparable ethical standards. No consents were obtained due to the retrospective nature of the study.

## TRANSPARENCY STATEMENT

I would like to confirm that the manuscript is an honest, accurate, and transparent account of the study being reported; that no important aspects of the study have been omitted; and that any discrepancies from the study as planned (and, if relevant, registered) have been explained.

## Data Availability

All data generated or analyzed during this study are included in this published article.
